# Effects of wood smoke particles from wood-burning stoves on the respiratory health of atopic humans

**DOI:** 10.1186/1743-8977-9-12

**Published:** 2012-04-30

**Authors:** Ingunn Skogstad Riddervold, Jakob Hjort Bønløkke, Anna-Carin Olin, Therese Koops Grønborg, Vivi Schlünssen, Kristin Skogstrand, David Hougaard, Andreas Massling, Torben Sigsgaard

**Affiliations:** 1 Department of Public Health, Section for Environmental and Occupational Medicine, Aarhus University, Aarhus, Denmark; 2 Department of Occupational and Environmental Medicine, Sahlgrenska University Hospital and Academy, Gothenburg, Sweden; 3 Department of Public Health, Section for Biostatistics, Aarhus University, Aarhus, Denmark; 4 Department of Clinical Biochemistry, Statens Serum Institute, Copenhagen, Denmark; 5 Department of Environmental Science, Aarhus University, Roskilde, Denmark; 6 Department of Public Health, Section for Environmental and Occupational Medicine, University of Aarhus, Bartholins Allé 2, Building 1260, DK-8000, Aarhus C, Denmark

**Keywords:** Air pollution, Controlled exposure, Wood smoke, Particles, Airway inflammation, Lung function, Humans

## Abstract

**Background:**

There is growing evidence that particulate air pollution derived from wood stoves causes acute inflammation in the respiratory system, increases the incidence of asthma and other allergic diseases, and increases respiratory morbidity and mortality. The objective of this study was to evaluate acute respiratory effects from short-term wood smoke exposure in humans. Twenty non-smoking atopic volunteers with normal lung function and without bronchial responsiveness were monitored during three different experimental exposure sessions, aiming at particle concentrations of about 200 μg/m^3^, 400 μg/m^3^, and clean air as control exposure. A balanced cross-over design was used and participants were randomly allocated to exposure orders. Particles were generated in a wood-burning facility and added to a full-scale climate chamber where the participants were exposed for 3 hours under controlled environmental conditions. Health effects were evaluated in relation to: peak expiratory flow (PEF), forced expiratory volume in the first second (FEV_1_), and forced vital capacity (FVC). Furthermore, the effects were assessed in relation to changes in nasal patency and from markers of airway inflammation: fractional exhaled nitric oxide (FENO), exhaled breath condensate (EBC) and nasal lavage (NAL) samples were collected before, and at various intervals after exposure.

**Results:**

No statistically significant effect of wood smoke exposure was found for lung function, for FENO, for NAL or for the nasal patency. Limited signs of airway inflammation were found in EBC.

**Conclusion:**

In conclusion, short term exposure with wood smoke at a concentration normally found in a residential area with a high density of burning wood stoves causes only mild inflammatory response.

## Background

Particulate air pollution can induce a major aggravation of respiratory symptoms and diseases. Because of this, the awareness of the impact of airborne particles related to different sources of air pollution, particularly fine and ultra fine particles, on human health is increasing. It is established that exposure to secondhand tobacco smoke particles during childhood increases the risk of asthma and other allergic diseases [1]. Growing evidence also indicates that particulate matter (PM) from diesel vehicle exhaust or concentrated ambient particles (CAPs) has the potential to cause or exacerbate asthma [2,3]. Researchers’ hypothesise that increased mortality can be associated with the particle levels in urban air [4-7]. Several studies have reported that especially the fine and ultrafine particles have an adverse effect on airways; and that children and asthmatics, among other vulnerable groups, may be at greater risk for developing adverse health effects of air pollutants [8].

Recent reviews have thoroughly discussed the relationship between wood smoke exposure and health effects [9-11]. It is well-established within air pollution research, that wood-burning stoves and fireplaces as well as agricultural and wild fires emit significant quantities of known health-damaging pollutants to both ambient and indoor air. The burning of wood gives rise to study pollutants like chlorinated dioxin, carbon monoxide (CO), methane, volatile organic compounds (VOC), nitrogen oxides (NO_x_), polycyclic aromatic hydrocarbons (PAH), and particulate matter (PM) [9,12]. These pollutants may trigger cough, throat and mucosal irritation, can cause acute inflammation in the respiratory system, and may contribute to an increased incidence of asthma and allergic diseases observed after prolonged exposure [8]. Approximately one-third of the world’s population and most of the rural households in developing countries still rely on unprocessed biomass fuels for cooking and heating [13]. Wood, dung and crop residues are typically burnt indoors on open fires or poorly functioning stoves, often causing extreme pollution levels indoors. In developing countries, these indoor pollution levels can be a serious threat to the health of especially women and children. Children are often carried on their mothers’ backs while cooking and therefore spend many hours breathing wood smoke particles and other related pollutants [14]. Some of the effects of wood smoke particle exposure are decreased pulmonary function and evidence of airway inflammation [8,15]. Furthermore, an increasing number of studies indicate a correlation between wood smoke exposure and lung diseases, such as acute respiratory infections (ARI) [16,17] and chronic obstructive pulmonary disease (COPD) [18-21]. Schei and colleagues showed that the prevalence of symptoms of asthma were higher in children from households that used open fires compared to those with improved stoves with chimneys [22].

The levels of respirable particles from wood burning in the outdoor environment in developed countries may be magnitudes lower compared to high exposures indoor in a rudimentary kitchen with poor ventilation. Still, there is increased concern about possible health effects as a consequence of wood smoke pollution due to the increasing use of wood burning. In recent years, exposure to fine and ultra fine airborne particles has been identified as an important factor affecting human health in the developed world [23-25], but the mechanisms underlying these effects are still unclear.

Recently, four experimental studies assessing comparable levels of wood smoke exposure on humans have been conducted. In an exposure study from Gothenburg, increasing alveolar NO and FENO_270_ (i.e. fraction of exhaled nitric oxide at exhalation flow of 270 ml/s) were found 3 hours after exposure, indicating inflammation in the lower respiratory system [15]. In a second study from Gothenburg the fraction of FENO also increased after wood smoke exposure [26]. This finding could not be confirmed in the study by Sehlstedt and colleagues, where NO levels were unaffected by wood smoke exposure, as were lung function parameters [27]. Ghio and colleagues reported from their human experiment that wood smoke exposure among other could be associated with pulmonary inflammation [28].

In the present study, we report the results from respiratory health measurements among atopics exposed to wood smoke in a controlled experiment. The hypothesis tested was that short-term exposure to wood smoke could induce acute signs of nasal and pulmonary inflammation. Other results from this study (coagulation and systemic inflammatory biomarkers) will be reported elsewhere.

## Results

**Figure 1:**
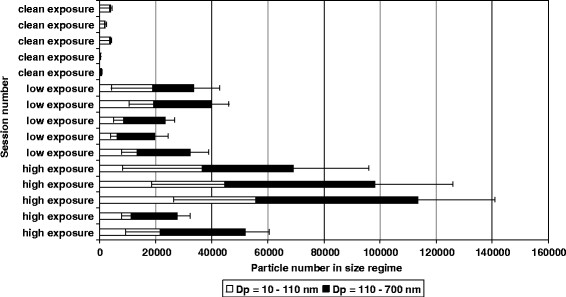
**The number of particles with diameters of Dp**_**10-110nm**_**and of Dp**_**110-700nm**_**during high, low, and clean air exposure is shown.** Negative error bars for the lower size regime N_10-110_ show one standard variation and positive error bars for the larger size regime N_110-700_ show also one standard variation of the measurement. Please note that concentration values for the clean exposure sessions are multiplied by a factor of 10 (in order to make the bars visible on the figure).

Figure 1 presents summary statistics on the achieved exposure levels. Table 1 presents the estimated changes from baseline to end of exposure (3½ h) and to 6 h for all the included outcomes, respectively. For the majority of the outcomes the estimated values for the change over time within each exposure are included in the confidence intervals (CI) for the other exposures, suggesting no differences in changes over time between exposures. None of the outcomes except from conductivity and nasal volume (vol_2-4_) seemed to be influenced by the RH% and the CO levels between the exposures. Therefore, all p-values, estimates and confidence intervals are based on the models not including these variables.

**Table 1:** Estimated changes from baseline (0 h) to end of exposure (3½ h) and to 6 h for all the respiratory outcomes

		**Clean air**				**Low wood smoke exposure**				**High wood smoke exposure**			
		**Change (0 h-3½ h)**		**Change (0 h-6 h)**		**Change (0 h-3½ h)**		**Change (0 h-6 h)**		**Change (0 h-3½ h)**		**Change (0 h-6 h)**	
**Variable category**	Unit	Mean	(CI)	Mean	(CI)	Mean	(CI)	Mean	(CI)	Mean	(CI)	Mean	(CI)
**Lung function**													
FEV1	Liters	**−0.01**	(−0.07 ; 0.05)	**0.00**	(−0.05 ; 0.06)	**0.03**	(−0.02 ; 0.09)	**0.04**	(−0.01 ; 0.10)	**0.02**	(−0.04 ; 0.07)	**0.06**	(0.00 ; 0.11)
FVC	Liters	**0.05**	(−0.09 ; 0.18)	**0.04**	(−0.09 ; 0.17)	**0.02**	(−0.11 ; 0.16)	**0.05**	(−0.08 ; 0.17)	**0.13**	(−0.01 ; 0.26)	**0.15**	(0.03 ; 0.27)
PEF	Liters/min	**−18.45**	(−34.93 ; -1.97)	**−15.30**	(−34.33 ; 3.73)	**−9.69**	(−25.75 ; 6.37)	**−5.28**	(−23.83 ; 13.27)	**−11.39**	(−27.10 ; 4.31)	**−6.64**	(−24.77 ; 11.49)
**Fractional exhaled NO**													
FENO50	ppb NO	**−2.14**	(−4.07 ; -0.21)	**N/A**	**N/A**	**−0.26**	(−2.14 ; 1.63)	**N/A**	**N/A**	**−1.60**	(−3.48 ; 0.29)	**N/A**	**N/A**
FENO270	ppb NO	**−0.38**	(−0.95 ; 0.18)	**N/A**	**N/A**	**0.07**	(−0.48 ; 0.62)	**N/A**	**N/A**	**−0.21**	(−0.76 ; 0.34)	**N/A**	**N/A**
**Exhaled breath condensate**													
Conductivity ^#^	μS/cm	**3.52**	(−14.00 ; 21.03)	**−5.54**	(−142.94 ; 131.86)	**−21.66**	(−38.91 ; -4.42)	**13.65**	(−120.27 ; 147.57)	**−2.80**	(−19.75 ; 14.15)	**−201.27**	(−331.98 ; -70.57)
pH		**0.25**	(0.01 ; 0.50)	**0.19**	(−0.14 ; 0.51)	**0.10**	(−0.14 ; 0.33)	**0.34**	(0.03 ; 0.66)	**0.02**	(−0.23 ; 0.25)	**−0.23**	(−0.54 ; 0.08)
8-Isoprostane	pg/ml	**0.12**	(−4.38 ; 4.61)	**−0.73**	(−11.05 ; 9.58)	**−6.16**	(−10.54 ; -1.78)	**−4.15**	(−13.92 ; 5.62)	**0.53**	(−4.08 ; 5.14)	**−7.40**	(−16.95 ; 2.14)
**Nasal lavage biomarkers**													
IL-1β	pg/ml	**−1.41**	(−4.81 ; 2.00)	**0.19**	(−2.83 ; 3.21)	**0.35**	(−2.97 ; 3.67)	**0.85**	(−2.10 ; 3.80)	**−1.14**	(−4.43 ; 2.14)	**2.51**	(−0.42 ; 5.44)
IL-6	pg/ml	**6.62**	(−0.41 ; 13.65)	**2.39**	(−5.69 ; 10.47)	**−1.90**	(−8.60 ; 4.80)	**−8.10**	(−15.85 ; -0.35)	**5.42**	(−1.14 ; 11.98)	**5.48**	(2.10 ; 13.06)
IL-8 ^#^	pg/ml	**0.86**	(−3.63 ; 5.36)	**2.92**	(−1.89 ; 7.74)	**2.40**	(−1.92 ; 6.73)	**0.70**	(−3.93 ; 5.33)	**−5.47**	(−9.72 ; -1.22)	**0.38**	(−4.16 ; 4.92)
IL-12 ^#^	pg/ml	**1.20**	(−0.81 ; 3.21)	**−0.75**	(−3.10 ; 1.61)	**−0.80**	(−2.71 ; 1.11)	**−2.20**	(−4.46 ; 0.06)	**1.86**	(−0.01 ; 3.73)	**1.48**	(−0.73 ; 3.69)
IL-18 ^#^	pg/ml	**−1.49**	(−6.00 ; 2.63)	**−6.00**	(−10.64 ; -1.35)	**−0.95**	(−4.88 ; 2.98)	**0.00**	(−4.46 ; 4.46)	**−3.21**	(−7.06 ; 0.63)	**−2.71**	(−7.07 ; 1.66)
**Nasal volume**													
VOl2-4 ^#^	ml	**0.20**	(0.01 ; 0.39)	**0.16**	(−0.04 ; 0.35)	**0.22**	(0.03 ; 0.41)	**0.20**	(0.01 ; 0.39)	**0.14**	(−0.05 ; 0.32)	**0.24**	(0.05 ; 0.42)

N/A (Not available, no measurement).

^#^Estimates based on the modified mixed model (not including the random patient*expo).

**Figure 2:**
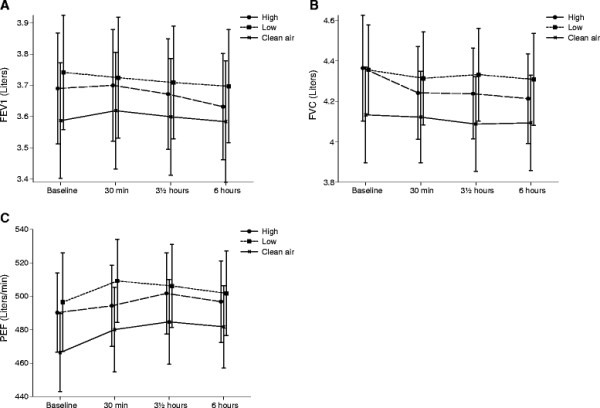
**Mean values of the different lung function measurements;** FEV1 (**A**), FVC (**B**) and PEF (**C**) at baseline, after 30 min of build-up exposure, at the end of exposure (3½ hours) and 6 h post exposure initiation for the 3 exposures to clean filtered air, low and high concentrations of wood smoke. Error bars represent +1 SEM.

**Figure 3:**
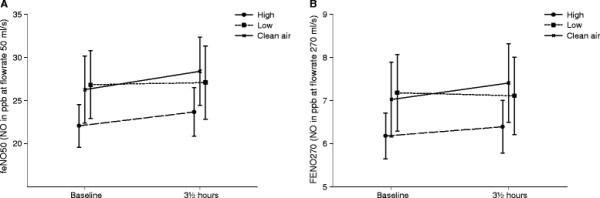
**Mean values of Fractional Exhale NO measurements:** FENO_50_ (**A**) and FENO_270_ (**B**) are shown at baseline and at the end of exposure (3½ hours) for the 3 exposures to clean filtered air, low and high concentrations of wood smoke. Error bars represent +1 SEM.

The results of the lung function measurements are presented in Figure 2. No statistically significant differences over time between the three exposures were found for any of the outcomes: PEF (p = 0.7453); FEV1 (p = 0.6283); and FVC (p = 0.8364). FENO levels at baseline and after exposure for each exposure are illustrated in Figure 3. No significant effect of exposure over time was found for FENO_50_ (p = 0.3578) or FENO_270_ (p = 0.5081).

**Figure 4:**
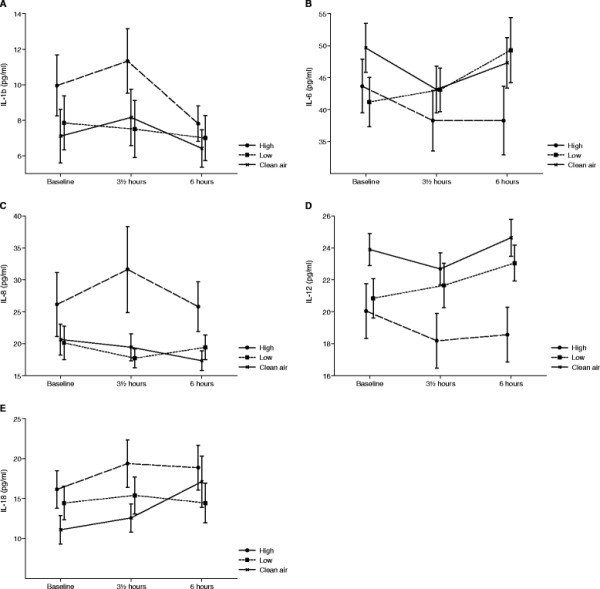
**Selected markers found in nasal lavage;** Selected markers are illustrated by mean values of IL-1β (**A**), IL-6 (**B**), IL-8 (**C**), IL-12 (**D**) and IL-18 (**E**) at baseline, at the end of exposure (3½ hours) and 6 h post exposure initiation for the 3 exposures to clean filtered air, low and high concentrations of wood smoke. Error bars represent +1 SEM.

Most of the measured nasal lavage cytokines did not show any variations related to time or exposure and the majority of the measurements were below the lower detection limit (LDL). This was true for IL-4, IL-5, IL-10, GM-CSF, IFN-γ, MCP-1, MIP-1α, RANTES, TGF-β1 and TNF-α and these data were therefore not analysed or presented graphically. The curves for the remaining nasal lavage cytokines are presented in Figure 4. Even though Figure 4 suggests cytokine levels responding to wood smoke exposure, none of the concentrations of the analysed biomarkers in nasal lavage were statistically significantly different when using the mixed model effect of exposure over time. (IL-1β: p = 0.3256; IL-6: p = 0.1133; IL-8: p = 0.0704; IL-12: p = 0.1663; IL-18: p = 0.2139).

**Figure 5:**
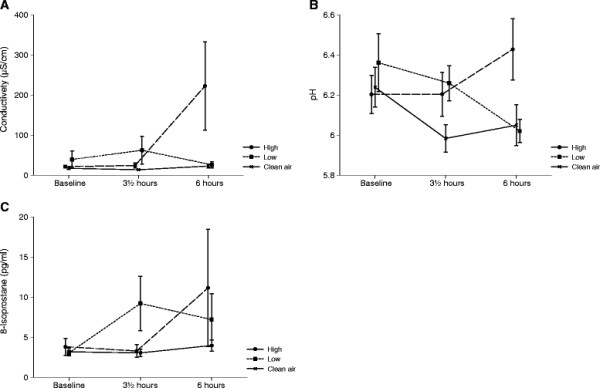
**Selected measurements obtained from the Exhaled Breath Condensate;** Selected measurements are shown as mean values of conductivity (**A**), pH (**B**) and 8-Isoprostane (**C**) at baseline, at the end of exposure (3½ hours) and 6 h post exposure initiation for the 3 exposures to clean filtered air, low and high concentrations of wood smoke. Error bars represent +1 SEM.

Curves for conductivity, pH and 8-isoprostane in EBC are shown in Figure 5. Conductivity was not significantly changed for exposure over time (p = 0.9998), but when RH% and levels of CO were included in the model as explaining variables the result reached statistical significance (p = 0.0228). For pH there was a statistical significant effect of exposure over time (p = 0.0331). The levels of 8-isoprostane were found not to be significant for exposure over time (p = 0.1795). Figure 4 shows a tendency to increased inflammation for the high wood smoke exposure 6 hours post exposure initiation.

**Figure 6:**
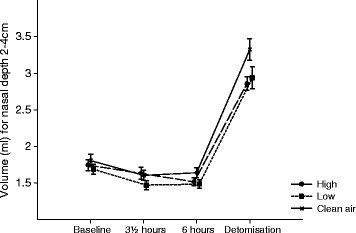
**Mean values of nasal patency (Vol**_**2-4 cm**_**) are illustrated;** at baseline, at the end of exposure (3½ hours) and 6 h post exposure initiation for the 3 exposures to clean filtered air, low and high concentrations of wood smoke. Detomisation represents the measurement after inhalation of nose drops. Error bars represent +1 SEM.

The nasal patency assessed by the nostril sum of the vol_2-4_ was not significantly affected by the exposure over time (p = 0.5452). The time pattern changes in nasal patency can be seen in Figure 6.

## Discussion

The aim of this study was to investigate whether short-term experimental wood smoke exposure induced inflammation in the airways of healthy atopic subjects. Despite the relatively high particle concentrations during the wood smoke exposure sessions and symptoms of airway mucosal irritation as reported in detail elsewhere [29], very few measurable effects were observed.

No statistically significant effect of wood smoke exposure was found for any of the lung function measurements, although there were non-significant indications of mild airway inflammation in the high exposure sessions regarding exhaled NO, airway inflammatory markers in NAL and nasal patency. The only outcomes that were found to be significantly associated to exposure over time were the conductivity and pH level measured in EBC.

The specific wood smoke exposure levels used in this study were chosen to have levels comparable with similar studies and to reveal information of the possible dose–response associations between wood smoke exposure and different health outcomes in atopics.

The dose during one day for the participants was in the order of 318 ug TSP/day during the day of the high concentration exposure lasting 3 h. Considering half the ambient air annually mean of 30ug/m^3^ in DK as the exposure for the rest of the day, an hourly inhaled amount of 400 L and a deposition fraction of 0.5. This compares to 75ug for a normal day in DK and 2,940 μg for a day in a citizen from Kenya who on average will be exposed for at least 1,000ug/m^3^ for 14 hours daily [30]. It follows, that the dosing we have used is approximately 4 times higher than a normal day in DK. However the dose was much lower than an average personal exposure in Kenya where the incidence of respiratory inflammation is high and related to the use of biomass for burning inside houses.

Changes in lung function as a method for measuring health effects of exposure to PM air pollution have been used for decades [8,31,32]. A study, completed during the wood-burning season, showed that FVC and FEV_1_ decreased in association with increases in PM air pollution in children [33]. Our findings of no observed changes in lung function is concordant with other experimental studies on wood smoke showing no significant changes in any lung function parameters investigated [27,34]. This lack of change in lung function has also been found in other studies where we observed airway inflammation [35], indicating that lung function measurements may be less sensitive than other measurements.

FENO has been suggested as a marker of airway inflammation and NO production has in some studies been reported to increase with high levels of air pollution [36,37]. Increased airway inflammation as assessed by FENO measurements has been associated with wood-burning PM in asthmatics [36]. Likewise, controlled exposure to wood smoke has been associated to increased levels in FENO_270_ 20 h after exposure [34]. Pietropauli et al., on the other hand, did not find any increase in distal NO production after exposure to ultrafine carbon particles [38]. In this experimental study neither FENO_50_ nor FENO_270_ increased after exposure to wood smoke. The fact that no influence on FENO_50_ was found indicated no significant inflammatory effect of the exposure on the conducting airways. No increase in the FENO_270_, was represented, to a larger extent than FENO_50_, NO deriving from the distal airways. Our findings are in accordance with a previous experimental study on effects of wood smoke exposure, in which no influence on FENO was found [27].

Our observation of very limited signs of inflammation, although weak, is in line with the two previous experimental wood smoke exposure studies [27,34]. Even though, no significant effects were found for any of the biomarkers measured in nasal lavage fluid, there seems to be a changing tendency of in the biomarker levels between exposures which seems to be most pronounced for the acute measurement just after exposure (see Figure 4). The general low concentrations of the different cytokines in the nasal lavage fluid may be explained by the method used where the fluid is collected after only 30 seconds. Furthermore, the fact that we flushed the nose twice before sampling in order to clear the nose of debris could possibly cause unwanted removal of the cells releasing the relevant biomarkers. However, the method is continuously being optimised [35,39]. It is obvious from earlier studies, that there is a downward shift in concentrations of all cytokines and cells from the first NAL to the subsequent ones. Hence inflammatory effects on nasal mucosa are seen as no deviation from baseline, whereas no inflammation results in a drop in cytokine level due to repeated lavage. We have further observed a great intra-individual variation in the pre-exposure concentration in the NAL [35]. The introduction of the pre-flushing was therefore set in place in order to bring down intra-individual variation and to create a “true” zero value. Figure 4B shows that there is a distinct different pattern for IL-6 after clean air and high exposure. This may be indicative of a slight inflammatory response after wood smoke exposure leading to an exhaustion of the IL-6 level in the nose as previously proposed by Krüger and colleagues [40].

Airway inflammation was further assessed by evaluating conductivity, pH, and levels of 8-isoprostane in exhaled breath condensate (EBC). The condensate pH is one of the most extensively studied nonspecific markers in EBC, and has been reported to be related to eosinophilic and neutrophilic inflammation of the airways [41]. However, a challenge with EBC samples is that concentrations of most markers of inflammation are near detection limits, resulting in high variability [12]. Only pH levels were found to be significantly affected by time-related exposure. Since electrical conductivity quantifies the ion content, it may be related to pH [42]. More comparable patterns for pH and conductivity were therefore expected. Isoprostanes appear as metabolites in tissue and plasma samples, which have undergone oxidative degradation during prolonged or improper storage. The fact that levels of 8-isoprostane were below the lower detection limit in the majority of our samples cannot be explained by prolonged or improper storage. It may therefore be considered if 8-isoprostane is a relevant marker to look for in EBC. As seen in Figure 5, changes in EBC seem to be most pronounced for the measurement 6 hours post exposure initiation. The presence of significant changes in the EBC only is probably a reflection of the compartment sampled. Compared to NAL EBC is sampled deeper in the airways, and therefore represents an area with a higher susceptibility to environmental challenge. The correction for RH and CO increased the significance of the EBC findings which is intriguing since we know that the ambient humidity influences the toxicity of the particles and the CO-content. The fact that no severe effects on the respiratory system were found is supported by other findings in this study. We observed borderline significant effects in the symptom index of “Weak Inflammatory Responses” and no significant effect of exposures were found for the symptom index of “Lower Respiratory Effects” [29].

Although health outcomes and exposure conditions are not directly comparable when studying respiratory outcomes in populations at large and in controlled exposures, no major effects were detectable during exposures lasting a few hours in healthy participants at rest or mildly exercising. Our findings are in concordance with other investigations studying acute effects of short-term wood smoke exposure [15,26-28].

During the study, the problem occurred that despite the fact that the exposure order was randomised, the baseline values during some exposures clearly deviate from others. Nevertheless, it is important to remember, that all participants participated in all exposure session types, and that the difference can therefore not be ascribed different treatments groups. We have no reasonable explanation for this variation and believe that we have done everything possible to prevent this variation by using the balanced randomised cross-over design, by pre-conditioning the participants before starting the experimental day, and by introducing pre-flushing of the nose. Considerable variations in both the between-participants and within-participants variation were present for most outcomes in this study. In a majority of the cases, the variation seemed to be larger than the possible effect, ruling out all possibilities of measurable effects from wood smoke exposure. It is however interesting that several graphs showed almost no differences between clean air exposure and low wood smoke exposure and only a difference between low and high exposure. Generally, it seems as though the variation is more pronounced for the high exposure sessions indicating huge differences in the responses to exposure to high levels of wood smoke. The observed variation may partly be caused by the investigated population. Atopy was expected to represent a homogenous study group, which we suspected to be more vulnerable and more directly respondent to wood smoke exposure. However, our findings suggest that atopics respond very differently to exposure, and therefore may be considered as a very heterogeneous population, unsuitable for investigating health effects from short-term exposure. This is supported by the fact that baseline levels for several outcomes varied greatly between exposure sessions and participants. We designed the study carefully to minimize random variation. We don’t believe that diurnal variation can explain the differences we find between exposure sessions since these were conducted at the same week days and in the same time of the day for all participants. Moreover, we tried to restrict the problematic activities of the participants during the preceding days in order to minimize this variation. Still we found variation in the baseline between days for the individuals, and this attenuates any responses even though the individual baseline is the basis and hence, controlled for in the mixed model analysis.

In this study multiple comparisons were made which increases the risk of finding false positives. According to Rothman and Greenland [43] multiple comparison adjustment (Bonferoni corrections) is not pertinent to this type of study. However, we are aware that this requires more caution in the data interpretation and conclusiveness of the study. However, we emphasize effects that might be biologically plausible or can be supported by similar findings in other studies.

The majority of the included outcomes were not indicative of inflammation, and where significance was observed these were marginal. The lack of significant findings may be explained by some natural human defense mechanisms that might cope with high wood smoke exposures for a limited exposure period.

On the other hand, based on the existing scientific evidence it is recognized that several of the included health outcomes may be related to wood smoke exposure why effects like these are biological plausible. In addition, similar findings of limited signs of airway inflammation are seen in most of the controlled human experiments. From our mixed model analyses we can exclude effect of learning and carry-over between exposure sessions and exclude potential effects of RH% and CO-pollutants. Compared to other conducted experimental wood smoke exposure studies this experiment included the highest number of participants and used the most optimal controlled design (randomized, blinded, balanced cross-over). Our own estimate suggest the dose administered to our participants was significantly lower than dose encountered by people in the third world on a daily basis, where an association to clinical inflammation has been shown. We believe that a true but very mild effect of the wood smoke exposure occurred in this study, chance is an alternative explanation, and therefore interpretation should be made with caution.

## Conclusions

In conclusion, the results of this study indicate that wood smoke, at least from the exposure situation under investigation, do not exert severe acute toxic effects, as no changes in lung function or nasal patency were observed. Only very mild inflammatory responses of inflammatory parameters were seen mainly in the central airways. Effects during prolonged wood smoke exposure or with exercise cannot be precluded with our current knowledge.

## Methods

### Design

This experimental study used a balanced cross-over design. The exposure sessions were carried out in groups of 4 participants randomised to the six possible exposure orders. All participants attended three different exposure sessions: clean air (<20 μg PM/m^3^) low particle concentration (~200 μg PM/m^3^) and high particle concentration (~400 μg PM/m^3^) for 3 hours. Each exposure session was separated by at least a 2-week period. The study was blinded and clean air exposures and wood smoke sessions were identical except for the air quality [29].

### Study population

Twenty non-smoking atopic volunteers (10 males, 10 females, mean age 25.1 years) with normal lung function and no bronchial hyper responsiveness completed the study. All participants underwent a standard medical assessment consisting of medical history and clinical examination. Atopy was determined by skin-prick testing to 10 common aeroallergens. Atopy was defined as a positive skin-prick tests (the mean of the longest diameter and the midpoint orthogonal diameter of the weal >3 mm) to one or more of the 10 common allergens. Bronchial hyper-responsiveness was measured using the method of Yan et al. [44] with De Vilbiss nebulisers connected to a device that operates on compressed air and produces a pressure pulse similar to that created by a hand [45], delivering cumulative dose of 2.49 mg metacholine bromide. Subjects whose FEV_1_ dropped by ≥ 20% of baseline FEV1 were considered as bronchial hyper responsive (BHR). Further exclusion criteria were a medical history of diseases, which could involve a risk for the participant or possibly influence the outcome measurements. Prior to each exposure session participants had to be free of infections or airway symptoms for at least 1 week and were not allowed to have taken any medication or drugs within the last 48 hours before exposure. Written consent was obtained from all participants and the study protocol was approved by The Aarhus County Human Study Review Board in accordance with the regulations for the protection of the participants (Ref. no. 20070097).

### Exposure facilities and exposure description

This study was conducted at the Section for Environmental and Occupational Medicine, Aarhus University. Exposure sessions took place under controlled conditions in a 79 m^3^ climate chamber optimised for experiments with gasses and particles as air pollutants. Environmental conditions (temperature and humidity) were monitored and kept constant throughout the experiment.

Wood-smoke was generated in a wood-stove facility using a Morsø wood stove (model 7110). Only beech-wood (standardised logs of approx. 1 kg (± 200 g) in mass with a relative humidity at 16-20%) was used in the stove and burned at a high temperature in order to achieve optimal and stable burning conditions. The wood smoke was aged in a pre-chamber and mixed with filtered outdoor air to reach the target concentration. After a transport and aging time of 5–10 min, the air reached the climate chamber causing the exposure. Combustion procedures were the same for all exposure sessions, but during clean air exposure the wood smoke inlet to the climate chamber was kept closed.

The exposure atmosphere was characterised by a mean temperature of 22.94°C for clean filtered air, 22.97°C for low PM exposure and 22.92°C for high PM exposure. The mean relative humidity was 22.04%, 33.97% and 32.92% for clean air, low and high PM exposure, respectively. For CO, the mean concentration was 0 ppm in the clean air exposure and 9.85 ppm and 16.05 ppm in the low and the high PM wood smoke exposures, respectively.

Size-fractioned particle sampling (TSP, PM_2.5_ and PM_10_) was obtained with stationary measurements. The TSP load was in the range 183–263 μg particles/m^3^ for the low exposure and 215–649 μg particles/m^3^ for the high exposure. The PM_2.5_ load was 165–303 μg particles/m^3^ for the low exposure and 205–662 μg particles/m^3^ for the high exposure. The range of the PM_10_ load were for low and high exposure 165–249 μg particles/m^3^ and 213–640 μg particles/m^3^, respectively. In clean air sessions, stationary PM samplers showed that the mean particle load was most often below the detection limit (<20 μg/m^3^). The particle number size distribution for the observed aerosols was monitored using a Differential Mobility Particle Sizer (DMPS) operating in a size range with particle diameters (Dp) of Dp = 10–700 nm [46]. During low and high PM exposures, the particle number size distribution generally resulted in a bimodal distribution, hinting that emitted particles from the wood combustion showed two chemically different fractions. Thus, the particle number in two size regimes N_10-110_ (10 nm < Dp < 110 nm) and N_110-700_ (110 nm < Dp < 700 nm) was determined. In Figure 1, the number of particles in size regimes with particle diameters of Dp_10nm–110nm_ and of Dp_110nm-700nm_ is presented for the low, the high and the clean air individual exposure sessions. Average number concentrations in N_10-110_ were about 33919 (± 17757) and in N_110-700_ about 38103 (± 17287) during high exposure sessions. In contrast, these values correspond to 13289 (± 5923) for N_10-110_ and 16397 (± 3051) for N_110-700_ during low exposure sessions. The fractionation of particles measured for the two size regimes was quite stable with regard to the different sessions. The average number N _10–110_ of particles within individual sessions varied only between 41 and 53 % and 32 and 57 % compared to the total number N_total_ of particles during the high and the low exposure sessions, respectively. Particle numbers in the respective size regimes were about a factor of 100 lower during clean air exposure sessions. Except for one exposure session, the total number of particles was generally about twice as high during high compared to low exposure sessions. Particles in the lower size regime N_10-110_ are assumed to have larger contribution from alkali salts as those in the larger size regime N_110-700_ are assumed to have higher contribution from carbonaceous aerosol. Further exposure details have been reported elsewhere [29].

All exposure sessions were conducted at the approximately same time of day to minimize the influence of diurnal variation in the outcome measurements. Participants entered the climate chamber and had a 30-min acclimatisation period with clean air prior to exposure. Following acclimatisation, approximately 30 min were used to build up the exposure, followed by 3 hours of maintained exposure. Participants were exposed at rest sitting at a desk. After exposure (3½ h) until the late follow-up measurements (6 h) participants stayed indoors at rest to minimize competing exposures to influence these measurements. The climate chamber was thoroughly cleaned before each exposure session and participants wore clean-suits to avoid unintended contamination in the chamber.

### Clinical measurements and biomarkers

Several measurements were carried out over time to assess respiratory and inflammatory effects. The outcomes were spirometry, fractional exhaled NO (FENO), nasal lavage (NAL), exhaled breath condensate (EBC) and nasal patency. Prior to each exposure session baseline measurements were obtained and follow-up measurements were carried out at selected time points. Time for initiation of exposure was set to time 0. Follow-up measurements were performed after 30 min (30 min ~ after building up the exposure) after additional 3 hours of maintained exposure (3½ hours ~ the end of exposure) and at 6 hours post exposure initiation (6 hours). All methods are standard methods used in our previous exposure studies [35,39,47,48]. For all outcomes the participants served as their own controls.

A MicroDL pocket spirometer (Micro Medical Limited, UK) was used to measure the flow/time profile of a full forced exhalation after maximal inhalation to obtain the peak expiratory flow (PEF). Testing was performed in accordance with the American Thoracic Society guidelines [49]. Electronically, a curve using the standards supplied by the Danish Society of Lung Physicians [50] was calculated and integrated to give predicted values of Forced Expiratory Volume in 1 sec (FEV_1_) and forced vital capacity (FVC).

Fractional exhaled nitric oxide (FENO) was measured using a chemiluminescence analyser (NIOX system; Aerocrine AB, Sweden). The following flow rates were used to assess different fractions of exhaled NO; 10 ml/s (FENO_10_), 50 ml/s (FENO_50_), 100 ml/s (FENO_100_), and 270 ml/s (FENO_270_). During the plateau phase an instant flow (±10%) and a mean flow (±5%) of the flow aimed at was accepted. All measurements were performed in duplicate according to the 2005 ATS/ERS recommendations after at least 1 hour of fasting. Prior to the study we considered calculating alveolar NO according to Tsoukias et al. [51]. However, only 70 out of our 120 measurements fulfilled the quality criteria of R^2^ ≥ 0.7, which is the reason not to present this data in this study. Consequently, only changes in FENO_50_ and FENO_270_ are reported here.

NAL samples were conducted from the participant sitting with a flexed neck. Through a nasal cork plug attached to a syringe, 5 ml of 0.9% sterile saline water (~37°C) was injected into the nostril. The saline water was held in the nasopharyngeal region for 30 sec and was then collected in a cup. The lavage was then repeated in the other nostril. For baseline measurements nasal lavage flush 1 and 2 were discharged in order to clean the nose from cellular debris and to receive a zero baseline, and only flush number 3 was analysed. Each nostril was flushed only once for the follow-up lavages. Each nasal lavage sample was transferred to a centrifuge tube, and the amount of fluid was determined by differential weighing and separated into a pellet and the supernatant. The samples were kept on ice during processing and the supernatant was kept frozen until analysis. The supernatant samples were analysed for analytes (Interleukin-1β (IL-1), IL-4, IL-5, IL-6, IL-8, IL-10, IL-12, IL-18, Tumor necrosis factor (TNF)-α, Interferon-γ (IFN-γ), granulocyte-macrophage colony stimulating factor (GM-CSF), transforming growth factor-1 (TGF-1), monocyte chemoattractant protein-1 (MCP-1), Macrophage inflammatory protein-1 (MIP-1), Regulated upon Activation, Normal T cell Expressed and Secreted (RANTES)) with an in-house assay as described by Skogstrand et al. [52]. In short, 50μL sample (undiluted nasal lavage) and 50μL of a suspension of capture-antibody-conjugated beads were mixed in plate wells. After 1.5 hours of incubation, the beads were washed twice and subsequently reacted for 1.5 hours with a mixture (50μL) of corresponding biotinylated detection antibodies, each diluted 1:1000. 50μL of streptavidin-phycoerythrin were added to the wells and the incubation was continued for an additional 30 min. Finally, the beads were washed twice and re-suspended in 125μL of buffer and analysed on the Luminex 100™ platform. All samples were measured in duplicate. Analytes measured in concentrations below their limit of detection were expressed as ½ of the lowest detection level in the statistical analyses.

EBC samples were collected with the participants sitting comfortably and breathing through a frozen cylinder for 10 min [53]. The participants were instructed to hold the cylinder upwards like a chimney pot or down to horizontal and never to hold it down, since saliva then might spill into the cylinder and contaminate the sample. The aluminum cylinders were kept in a −20°C freezer for at least 3 hours before the sampling, with the insulation in place. After the 10 min period, the cylinder was emptied into a small plastic cup and the condensate was further transferred to an Eppendorph tube. The tube was stored in a −80°C freezer until the analyses were performed. EBC samples were analysed for pH, electrical conductivity and 8-isoprostane. Due to the very limited condensate volume pH and conductivity were measured directly in the samples. Conductivity was measured with a WTW ino Lab Cond 730 (with an electrode WTW D82362 Weilheim Type LDM/S). For measuring the pH-values of the samples a pH meter (WTW pH 330i with a Hamilton minitrode) was used. The 8-Isoprostane analysis kit used was 8-isoprostane EIA Kit (catalog no. 516351, Cayman Chemical Company) with the following limits of detection: 80% B/B_0_: 2.7 pg/ml and sensitivity: 50% B/B_0_: 10 pg/ml.

Acoustic rhinometry was used to assess the nasal cross sectional area and volume [54]. The left and right nasal cavity were studied alternatively until three reproducible measurements were obtained. The minimum cross sectional cavity area was calculated from the means of the measurements. By integration of the area-distance curve, the sum of the volume 2 to 4 (vol_2-4_) from the nostril was determined on both sides. Acoustic rhinometry was as a rule performed before nasal lavage to avoid influences of the nose flushing on the nasal volume.

### Statistics

For the outcomes PEF, FEV1, FVC, FE_NO50_, FE_NO270_, IL-1β, IL-6, pH and vol_2-4_, a mixed model was fitted using SAS (SAS 9.2, SAS Institute Inc., USA). In the analyses class variables for exposure status (clean air, low exposure, high exposure) were used. Exposure, time, the interaction between exposure and time, learning effect, and carry-over effect were included as fixed effects. Patient, the interaction between patient and exposure, and the interaction between patient and time were included as random effects. Furthermore, the analyses were conducted including CO levels (continuous) and RH levels (continuous) to see whether these variables affected the results. Due to flooring-problems and a high number of zeros in the data sets the complex mixed model could not directly be fitted for IL-8, IL-12, IL-18, conductivity and vol_2-4_. By leaving out one of the model parameters of least importance (the random effect of the interaction between patient and exposure) the modified model could be fitted for these outcomes. A significance level of 0.05 was used in all analyses. The mixed models were used to detect statistical significant effects of exposure over time for all the included outcomes and to calculate estimates for the change (difference) from baseline to end of exposure (3½ h) and 6 h, respectively. The time courses of the health effects are described by mean comparisons relevant to the time of measurements, including the standard errors of the means (SEM) to show the underlying data variation.

## Competing interests

The authors declare that they have no competing interests.

## Authors’ contributions

**ISR** has contributed substantially to the completion of the study, acquisition, analysis and interpretation of the data, and has been the prime mover in relation to writing the manuscript. **JHB** has contributed substantially to the design, the completion of the study, the statistical analyses and been involved in drafting the manuscript. **A-CO** provided the equipment for the FENO measurements and contributed with the interpretation of the data. **TKG** contributed substantially with the statistical models and analyses. **VS** contributed to the design, the completion of the study and critically revising the manuscript for important intellectual content. **KS** and **DH** have contributed with the sample analysis of inflammatory markers. **AM** has provided equipment for the exposure measurements, contributed to the execution of the measurements and the analysis and interpretation of the exposure data. **TS** contributed substantially to the concept, the design and the completion of the study, the statistical analyses and critically revising the manuscript for important intellectual content. All authors have read and approved the final manuscript.
